# Supplementary material for: Metabolic phenotyping reveals an emerging role of ammonia abnormality in Alzheimer’s disease
Source: Nat Commun. 2024 May 7;15:3796. doi: 10.1038/s41467-024-47897-y (PMC11076546; doi:10.1038/s41467-024-47897-y)
Supplement: Supplementary file 1 — Supplementary Information [file 41467_2024_47897_MOESM1_ESM.pdf]

Supplementary information

Metabolic phenotyping reveals an emerging role of ammonia abnormality in Alzheimer's disease

Tianlu Chen[1#], Fengfeng Pan[2#], Qi Huang[3#], Guoxiang Xie[4], Xiaowen Chao[1], Lirong Wu[1], Jie Wang[3], Liang Cui[2], Tao Sun[1], Mengci Li[1], Ying Wang[2], Yihui Guan[3], Xiaojiao Zheng[1], Zhenxing Ren[1], Yuhuai Guo[1], Lu Wang[5], Kejun Zhou[4], Aihua Zhao[1], Qihao Guo[2*], Fang Xie[3*], Wei Jia[1,5*]

1 Center for Translational Medicine and Shanghai Key Laboratory of Diabetes Mellitus, Shanghai Sixth People's Hospital Affiliated to Shanghai Jiao Tong University School of Medicine, Shanghai 200233, China

2 Department of Gerontology, Shanghai Sixth People's Hospital Affiliated to Shanghai Jiao Tong University School of Medicine, Shanghai, 200233, China.

3 Department of Nuclear Medicine & PET Center, Huashan Hospital, Fudan University, Shanghai, 200040, China

4 Human Metabolomics Institute, Inc., Shenzhen 518109, China

5 School of Chinese Medicine, Hong Kong Baptist University, Kowloon Tong 999077, Hong Kong, China.

Contents

Table S1. Quantified metabolites 3
Figure S1. Fold changes of concentration summations of metabolite types in SCD, MCI, and AD relative to CN. 9
Table S2. The computational formula and biological meaning of 34 extended metabolic features. 10
Figure S2. Associations of 13 metabolic features and cognition scores in stratified participants. 12
Table S3. Characteristics of age-matched sub-population. 13
Figure S3. Performances of the 13 identified features in age-matched sub-populations. 14
Table S4. Metabolite measurement information of 8 validation data sets. 16
Table S5. Population characteristics of 8 validation data sets. 17
Figure S4 The number of overlapped participants between ADNI studies. 19
Figure S5. The meta-analysis forest plots of identified features on their standardized mean differences of CN and AD. 20
Figure S6. The meta-analysis forest plots of identified features on their Partial Spearman correlation coefficients with global cognition. 25
Methods 30
Discussion 35
References 37

Table S1. Quantified metabolites

index	name	HMDB	type
1	1-Methylhistidine	HMDB0000001	Amino Acids
2	Beta-Alanine	HMDB0000056	Amino Acids
3	Creatine	HMDB0000064	Amino Acids
4	L-Tyrosine	HMDB0000158	Amino Acids
5	L-Phenylalanine	HMDB0000159	Amino Acids
6	L-Alanine	HMDB0000161	Amino Acids
7	L-Proline	HMDB0000162	Amino Acids
8	L-threonine	HMDB0000167	Amino Acids
9	L-Asparagine	HMDB0000168	Amino Acids
10	L-Histidine	HMDB0000177	Amino Acids
11	L-Lysine	HMDB0000182	Amino Acids
12	L-Serine	HMDB0000187	Amino Acids
13	Ornithine	HMDB0000214	Amino Acids
14	Sarcosine	HMDB0000271	Amino Acids
15	L-Arginine	HMDB0000517	Amino Acids
16	glutamine	HMDB0000641	Amino Acids
17	Homocitrulline	HMDB0000679	Amino Acids
18	L-Methionine	HMDB0000696	Amino Acids
19	L-Pipecolic acid	HMDB0000716	Amino Acids
20	4-Hydroxyproline	HMDB0000725	Amino Acids
21	Citrulline	HMDB0000904	Amino Acids
22	L-Tryptophan	HMDB0000929	Amino Acids
23	5-Aminolevulinic acid	HMDB0001149	Amino Acids
24	Methylcysteine	HMDB0002108	Amino Acids
25	N-Acetylserine	HMDB0002931	Amino Acids
26	3-Aminoisobutanoic acid	HMDB0003911	Amino Acids
27	Phenylacetylglutamine	HMDB0006344	Amino Acids
28	L-Isoleucine	HMDB0000172	BCAAs
29	L-Leucine	HMDB0000687	BCAAs
30	L-Valine	HMDB0000883	BCAAs
31	glutamate	HMDB0000148	excitatory neurotransmitters
32	L-Aspartic acid	HMDB0000191	excitatory neurotransmitters
33	Pyroglutamic acid	HMDB0000267	excitatory neurotransmitters
34	N-Acetyl-L-aspartic acid	HMDB0000812	excitatory neurotransmitters
35	Dimethylglycine	HMDB0000092	inhibitory neurotransmitters

36	GABA	HMDB0000112	inhibitory neurotransmitters
37	Glycine	HMDB0000123	inhibitory neurotransmitters
38	Acetylglycine	HMDB0000532	inhibitory neurotransmitters
39	2-Phenylglycine	HMDB0002210	inhibitory neurotransmitters
40	TCA	HMDB0000036	Bile Acids
41	GCA	HMDB0000138	Bile Acids
42	7_DHCA	HMDB0000391	Bile Acids
43	CDCA	HMDB0000518	Bile Acids
44	CA	HMDB0000619	Bile Acids
45	DCA	HMDB0000626	Bile Acids
46	GDCA	HMDB0000631	Bile Acids
47	GCDCA	HMDB0000637	Bile Acids
48	isoDCA	HMDB0000686	Bile Acids
49	GLCA	HMDB0000698	Bile Acids
50	GUDCA	HMDB0000708	Bile Acids
51	HCA	HMDB0000760	Bile Acids
52	LCA	HMDB0000761	Bile Acids
53	TUDCA	HMDB0000874	Bile Acids
54	TDCA	HMDB0000896	Bile Acids
55	UDCA	HMDB0000946	Bile Acids
56	TCDCA	HMDB0000951	Bile Acids
57	GHCA	HMDB0240607	Bile Acids
58	NorCA	Norcholic acid	Bile Acids
59	Glyceric acid	HMDB0000139	Carbohydrates
60	Gluconolactone	HMDB0000150	Carbohydrates
61	N-Acetylneuraminic acid	HMDB0000230	Carbohydrates
62	Galactonic acid	HMDB0000565	Carbohydrates
63	Erythronic acid	HMDB0000613	Carbohydrates
64	Glucaric acid	HMDB0000663	Carbohydrates
65	Ribonic acid	HMDB0000867	Carbohydrates
66	Threonic acid	HMDB0000943	Carbohydrates
67	Tartaric acid	HMDB0000956	Carbohydrates
68	D-Xylose	HMDB0000098	sugars
69	D-Glucose	HMDB0000122	sugars
70	D-Maltose	HMDB0000163	sugars
71	D-Fructose	HMDB0000660	sugars
72	Rhamnose	HMDB0000849	sugars
73	D-Xylulose	HMDB0001644	sugars
74	L-Carnitine	HMDB0000062	Carnitines
75	L-Acetylcarnitine	HMDB0000201	Carnitines

76	Palmitoylcarnitine	HMDB0000222	Carnitines
77	2-Methylbutyroylcarnitine	HMDB0000378	Carnitines
78	Decanoylcarnitine	HMDB0000651	Carnitines
79	Isovalerylcarnitine	HMDB0000688	Carnitines
80	Hexanoylcarnitine	HMDB0000756	Carnitines
81	Octanoylcarnitine	HMDB0000791	Carnitines
82	Propionylcarnitine	HMDB0000824	Carnitines
83	Malonylcarnitine	HMDB0002095	Carnitines
84	Dodecanoylcarnitine	HMDB0002250	Carnitines
85	Oleoylcarnitine	HMDB0005065	Carnitines
86	Tetradecanoylcarnitine	HMDB0005066	Carnitines
87	Linoleyl carnitine	HMDB0006469	Carnitines
88	Valerylcarnitine	HMDB0013128	Carnitines
89	Glutarylcarnitine	HMDB0013130	Carnitines
90	Methylmalonylcarnitine	HMDB0013133	Carnitines
91	3-Hydroxyisovalerylcarnitine	HMDB0061189	Carnitines
92	O-Adipoylcarnitine	HMDB0061677	Carnitines
93	4-Methylhexanoic acid	4-Methylhexanoic acid	Fatty Acids
94	Oleic acid	HMDB0000207	Fatty Acids
95	2-Hydroxy-3-methylbutyric acid	HMDB0000407	Fatty Acids
96	Citramalic acid	HMDB0000426	Fatty Acids
97	Adipic acid	HMDB0000448	Fatty Acids
98	Capric acid	HMDB0000511	Fatty Acids
99	5Z-Dodecenoic acid	HMDB0000529	Fatty Acids
100	3-Methyladipic acid	HMDB0000555	Fatty Acids
101	Dodecanoic acid	HMDB0000638	Fatty Acids
102	Heptanoic acid	HMDB0000666	Fatty Acids
103	Linoleic acid	HMDB0000673	Fatty Acids
104	Azelaic acid	HMDB0000784	Fatty Acids
105	Sebacic acid	HMDB0000792	Fatty Acids
106	Myristic acid	HMDB0000806	Fatty Acids
107	Pimelic acid	HMDB0000857	Fatty Acids
108	Suberic acid	HMDB0000893	Fatty Acids
109	Tridecanoic acid	HMDB0000910	Fatty Acids
110	Undecanoic acid	HMDB0000947	Fatty Acids
111	Arachidonic acid	HMDB0001043	Fatty Acids
112	Alpha-Linolenic acid	HMDB0001388	Fatty Acids
113	2-Hydroxycaproic acid	HMDB0001624	Fatty Acids
114	Methylsuccinic acid	HMDB0001844	Fatty Acids

115	Docosapentaenoic acid (22n-6)	HMDB0001976	Fatty Acids
116	2-Hydroxy-2-methylbutyric acid	HMDB0001987	Fatty Acids
117	Eicosapentaenoic acid	HMDB0001999	Fatty Acids
118	Myristoleic acid	HMDB0002000	Fatty Acids
119	2,2-Dimethylsuccinic acid	HMDB0002074	Fatty Acids
120	Docosahexaenoic acid	HMDB0002183	Fatty Acids
121	Adrenic acid	HMDB0002226	Fatty Acids
122	Dihomo-gamma-linolenic acid	HMDB0002925	Fatty Acids
123	Gamma-Linolenic acid	HMDB0003073	Fatty Acids
124	Palmitoleic acid	HMDB0003229	Fatty Acids
125	Docosapentaenoic acid (22n-3)	HMDB0006528	Fatty Acids
126	Ricinoleic acid	HMDB0034297	Fatty Acids
127	12-hydroxystearic acid	HMDB0061706	Fatty Acids
128	9E-tetradecenoic acid	HMDB0062248	Fatty Acids
129	Caprylic acid	HMDB0000482	Fatty Acids
130	2-Hydroxybutyric acid	HMDB0000008	Organic Acids
131	Alpha-ketoisovaleric acid	HMDB0000019	Organic Acids
132	cis-Aconitic acid	HMDB0000072	Organic Acids
133	Citric acid	HMDB0000094	Organic Acids
134	Glycolic acid	HMDB0000115	Organic Acids
135	Guanidoacetic acid	HMDB0000128	Organic Acids
136	Fumaric acid	HMDB0000134	Organic Acids
137	L-Malic acid	HMDB0000156	Organic Acids
138	Maleic acid	HMDB0000176	Organic Acids
139	L-Lactic acid	HMDB0000190	Organic Acids
140	Isocitric acid	HMDB0000193	Organic Acids
141	Methylmalonic acid	HMDB0000202	Organic Acids
142	Oxoglutaric acid	HMDB0000208	Organic Acids
143	Oxoadipic acid	HMDB0000225	Organic Acids
144	Pyruvic acid	HMDB0000243	Organic Acids
145	Succinic acid	HMDB0000254	Organic Acids
146	3-Hydroxybutyric acid	HMDB0000357	Organic Acids
147	3-Methyl-2-oxovaleric acid	HMDB0000491	Organic Acids
148	D-2-Hydroxyglutaric acid	HMDB0000606	Organic Acids
149	Glutaconic acid	HMDB0000620	Organic Acids
150	Glutaric acid	HMDB0000661	Organic Acids

151	Malonic acid	HMDB0000691	Organic Acids
152	Hydroxypropionic acid	HMDB0000700	Organic Acids
153	Alpha-Hydroxyisobutyric acid	HMDB0000729	Organic Acids
154	Benzoic acid	HMDB0001870	Organic Acids
155	Oxalic acid	HMDB0002329	Organic Acids
156	Quinic acid	HMDB0003072	Organic Acids
157	Ketoleucine	HMDB0000695	Organic Acids
158	p-Hydroxyphenylacetic acid	HMDB0000020	others
159	Homovanillic acid	HMDB0000118	others
160	Indoleacetic acid	HMDB0000197	others
161	Phenylpyruvic acid	HMDB0000205	others
162	Phenylacetic acid	HMDB0000209	others
163	Ortho-Hydroxyphenylacetic acid	HMDB0000669	others
164	Indolelactic acid	HMDB0000671	others
165	Mandelic acid	HMDB0000703	others
166	Hippuric acid	HMDB0000714	others
167	Glycylproline	HMDB0000721	others
168	Hydroxyphenyllactic acid	HMDB0000755	others
169	Hydrocinnamic acid	HMDB0000764	others
170	Phenyllactic acid	HMDB0000779	others
171	Salicyluric acid	HMDB0000840	others
172	Phthalic acid	HMDB0002107	others
173	Imidazolepropionic acid	HMDB0002271	others
174	3-Indolepropionic acid	HMDB0002302	others
175	3-(3-Hydroxyphenyl)-3-hydroxypropanoic acid	HMDB0002643	others
176	N-Methylnicotinamide	HMDB0003152	others
177	gamma-Glutamylalanine	HMDB0006248	others
178	2-Phenylpropionate	HMDB0011743	others
179	Indolepyruvate	HMDB0060484	others
180	Butyric acid	HMDB0000039	SCFAs
181	Acetic acid	HMDB0000042	SCFAs
182	Propionic acid	HMDB0000237	SCFAs
183	Caproic acid	HMDB0000535	SCFAs
184	Isocaproic acid	HMDB0000689	SCFAs
185	Isovaleric acid	HMDB0000718	SCFAs
186	3-Hydroxyisovaleric acid	HMDB0000754	SCFAs

187	Valeric acid	HMDB0000892	SCFAs
188	Isobutyric acid	HMDB0001873	SCFAs
189	Ethylmethylacetic acid	HMDB0002176	SCFAs

BCAA: branched-chain amino acid;
SCFA: short-chain fatty acid

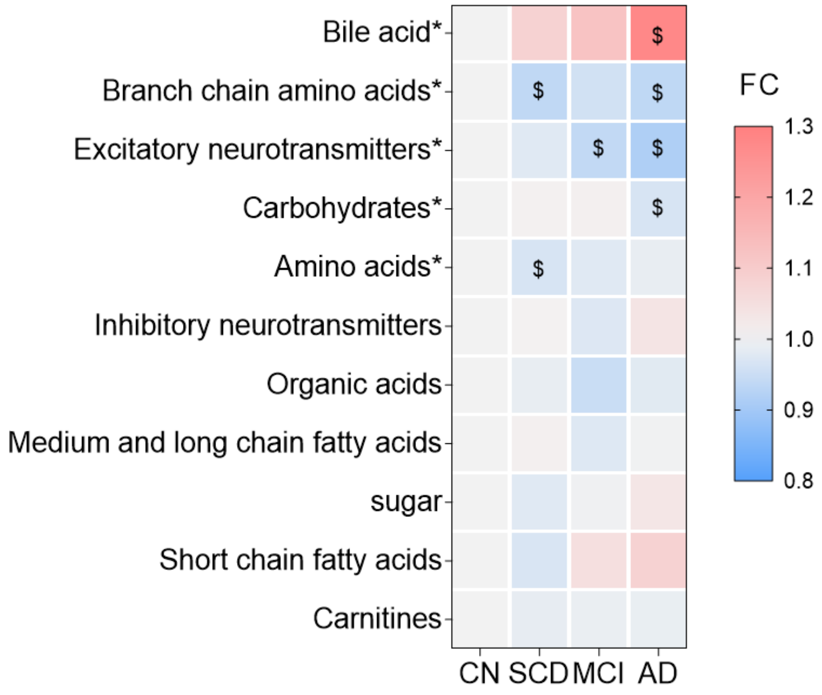

Figure S1. Fold changes of concentration summations of metabolite types in SCD, MCI, and AD relative to CN.

* indicates ANOVA FDR<0.05 comparing NC, SCD, MCI, and AD. $ indicates dunnett post hoc p<0.05 comparing to CN. All p values are two-sided. Metabolite types are ordered by ANOVA FDR values.

Table S2. The computational formula and biological meaning of 34 extended metabolic features.

index	name	Biological meaning (KEGG K number or computational formula)	type
1	BCAAs	concentration of total BCAAs (valine+leucine+isoleucine)	summation
2	glutamate/GABA	activity of glutamate decarboxylase (K01580)	ratio
3	glutamate/glutamine	activity of glutamine synthetase, glutamate synthase (NADH), and glutaminase (K01915, K00264, and K01425)	ratio
4	asparate/N-acetyl-L-aspartate	activity of aspartate N-acetyltransferase and aspartoacylase (K18309 and K01437)	ratio
5	aspartare/asparagine	activity of asparagine synthase (glutamine-hydrolysing), aspartate--ammonia ligase, and glutamin-(asparagin-)ase (K01953, K01914, K05597)	ratio
6	glutamate/oxoglutarate	activity of glutamate synthase (NADPH) large chain (K00265)	ratio
7	TBA	total BAs (concentration summation of 19 BAs), plasma BA pool	summation
8	ConBA	total conjugated BAs (GCA+TCA+GCDCA+TCDCA+GDCA+TDCA+GUDCA+TUDCA+GLCA), BA pool structure	summation
9	UnconBA	total unconjugated BAs (CA+CDCA+DCA+UDCA+LCA), BA pool structure	summation
10	PriBA	total primary BAs (CA+CDCA+GCA+GCDCA+TCA+TCDCA), BA pool structure	summation
11	SecBA	total secondary BAs (DCA+UDCA+LCA+GDCA+TDCA+GUDCA+TUDCA+GLCA), BA pool structure	summation
12	Pri/Sec	ratio of primary and secondary BAs, BA pool structure	ratio
13	Con/Uncon	ratio of conjugated and unconjugated BAs, BA pool structure	ratio
14	CA+CDCA	total primary unconjugated BAs, BA pool structure	summation
15	CA/CDCA	ratio of 2 primary unconjugated BAs, balance of classical and alternative pathway of BA metabolism	ratio
16	TCA/CDCA	liver enzymatic (including the bile acid -CoA: amino acid N-acyltransferase, K00659 and sterol 12-alpha-hydroxylase, K07431) activities and gut microbiome function (bile salt hydrolase, K01442)	ratio
17	GCA/CDCA	liver enzymatic (including the bile acid -CoA: amino acid N-acyltransferase, K00659 and sterol 12-alpha-	ratio

		hydroxylase, K07431) activities and gut microbiome function (bile salt hydrolase, K01442)	
18	DCA/CA	gut microbiome function (bile acid 7-alpha hydroxylation including K15868, K15870, K15872, K15871 and K15873)	ratio
19	GDCA/CA	liver enzymatic (bile acid -CoA: amino acid N-acyltransferase, K00659) activities and gut microbiome function (bile salt hydrolase, K01442; bile acid 7-alpha hydroxylation including K15868, K15870, K15872, K15871 and K15873)	ratio
20	TDCA/CA	liver enzymatic (bile acid -CoA: amino acid N-acyltransferase, K00659) activities and gut microbiome function (bile salt hydrolase, K01442; bile acid 7-alpha hydroxylation including K15868, K15870, K15872, K15871 and K15873)	ratio
21	LCA/CDCA	gut microbiome function (bile acid 7-alpha hydroxylation including K15868, K15870, K15872, K15871 and K15873)	ratio
22	GLCA/CDCA	liver enzymatic (bile acid -CoA: amino acid N-acyltransferase, K00659) activities and gut microbiome function (bile salt hydrolase, K01442; bile acid 7-alpha hydroxylation including K15868, K15870, K15872, K15871 and K15873)	ratio
23	UDCA/CDCA	gut microbiome function (7alpha/beta-HSDH, K00076/K23231)	ratio
24	GUDCA/CDCA	liver enzymatic (bile acid -CoA: amino acid N-acyltransferase, K00659) activities and gut microbiome function (bile salt hydrolase, K01442; 7alpha/beta-HSDH, K00076/K23231)	ratio
25	TUDCA/CDCA	liver enzymatic (bile acid -CoA: amino acid N-acyltransferase, K00659) activities and gut microbiome function (bile salt hydrolase, K01442; 7alpha/beta-HSDH, K00076/K23231)	ratio
26	GLCA/LCA	gut microbiome function (bile salt hydrolase, K01442)	ratio
27	TCA/CA	gut microbiome function (bile salt hydrolase, K01442)	ratio
28	TCDCA/CDCA	gut microbiome function (bile salt hydrolase, K01442)	ratio
29	TDCA/DCA	gut microbiome function (bile salt hydrolase, K01442)	ratio
30	TUDCA/UDCA	gut microbiome function (bile salt hydrolase, K01442)	ratio
31	GCA/CA	gut microbiome function (bile salt hydrolase, K01442)	ratio
32	GCDCA/CDCA	gut microbiome function (bile salt hydrolase, K01442)	ratio
33	GDCA/DCA	gut microbiome function (bile salt hydrolase, K01442)	ratio
34	GUDCA/UDCA	gut microbiome function (bile salt hydrolase, K01442)	ratio

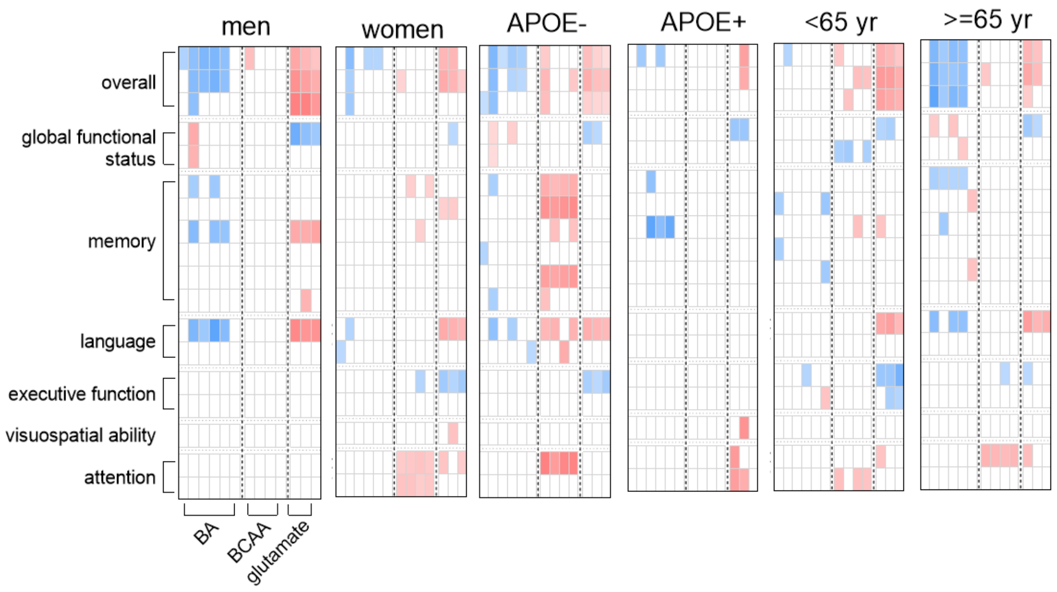

Figure S2. Associations of 13 metabolic features and cognition scores in stratified participants.

Cell color indicates correlation coefficient from Partial Spearman analysis adjusting age, sex, BMI, education year, and *APOE*-ε4 (red: positive; blue: negative; blank: p>=0.05; two-sided). The order of cognition test scores is consistent with that of main text figure 3b.

Table S3. Characteristics of age-matched sub-population.

Characteristic	ALL (n=991)	CN (n=259)	SCD (n=141)	MCI(n=225)	AD (n=366)
Age (yr)	69.7+6.5<br>[60,89]<br>69(65,74)	69.6+4.6<br>[64,84]<br>69(66,73)	69.4+4.7<br>[63,81]<br>69(65,72)	69.5+6.0<br>[60,86]<br>69(65,74)	69.9+8.4<br>[60,89]<br>70(65,76)
Sex (Men%)	37.8%	40.2%	35.5%	35.7%	38.4%
BMI (kg/m^2)	23.2+3.3*<br>[13.7,33.8]<br>23.1(21.0,25.3)	23.6+3.2<br>[15.4,33.8]<br>23.5(21.5,25.3)	23.5+3.1<br>[16.4,30.2]<br>23.5(21.2,25.9)	23.1+3.4<br>[15.5,33.2]<br>23.0(20.8,25.2)	22.9+3.4$^{\#}$<br>[13.7,31.1]<br>22.7(20.7,25.1)
Education(yr)	11.0+3.2*<br>[6, 22]<br>11(9,13)	11.8+3.1<br>[6, 20]<br>12(9,14)	11.6+3.2<br>[6,18]<br>12(9,14)	11.1+3.0$^{\#}$<br>[6, 22]<br>11(9,13)	10.1+3.2$^{\#}$<br>[6, 19]<br>10(7.2,12)
APOE-ε4 carrier %[a]	31.9%*	17.4%	17.7%	31.6%$^{\#}$	47.8%$^{\#}$
PET acceptance(%)[b]	28.3%*	36.3%	39.7%	28.4%	18.0%$^{\#}$
Brain Aβ+(%)[c]	34.3%	17.0%	19.6%	32.8%	72.7%
MMSE	23.4+6.0*<br>[10,30]<br>26(20,28)	28.0+1.7<br>[21,30]<br>28(27,29)	27.4+1.8$^{\#}$<br>[21,30]<br>28(26,29)	26.3+2.0$^{\#}$<br>[15,30]<br>27(25,28)	16.9+4.7$^{\#}$<br>[10,27]<br>18(12,21)
ACEIII-CV	64.9+18.7*<br>[10,97]<br>69(54,79)	80.8+7.9<br>[60,97]<br>81.5(76,87)	77.1+7.7$^{\#}$<br>[60,95]<br>77(72,82)	69.8+8.4$^{\#}$<br>[50,94]<br>71(64,75.2)	45.6+14.7$^{\#}$<br>[10,77]<br>48(36,58)
MoCA-BC	22.1+4.9*<br>[10,30]<br>23(19,26)	25.6+2.6<br>[20,30]<br>26(24,27)	24.0+3.0$^{\#}$<br>[17,29]<br>24(22,27)	21.7+3.3$^{\#}$<br>[15,30]<br>22(20,24)	15.3+3.3$^{\#}$<br>[10,22]<br>15(13,18)

Data are presented as mean+S.D., [minimum, maximum], and median (IQR), or percentage. * indicates Chi-squared test, analysis of variance, or Kruskal–Wallis test FDR<0.05 when comparing 4 groups (adjusted by Benjamini and Hochberg). # indicates Chi-squared test, student's t-test or Mann-Whitney test FDR<0.05 when compared to CN (adjusted by Benjamini and Hochberg). C-PAS: Chinese Preclinical Alzheimer's Disease Study; CN: cognitively normal; AD: Alzheimer's disease; SCD: subjective cognitive decline; MMSE: Mini-Mental State Examination; ACEIII-CV: Chinese version of Addenbrooke's cognitive examination-III; MoCA-BC: Chinese version of Montreal Cognitive Assessment-Basic. a: the percentage of *APOE*-ε4 carriers. b: the percentage of the participants that accepted brain PET test. c: the percentage of participants with positive Aβ (defined through visual rating following the guidelines for interpreting amyloid PET) in those underwent the brain AV45-PET scans.

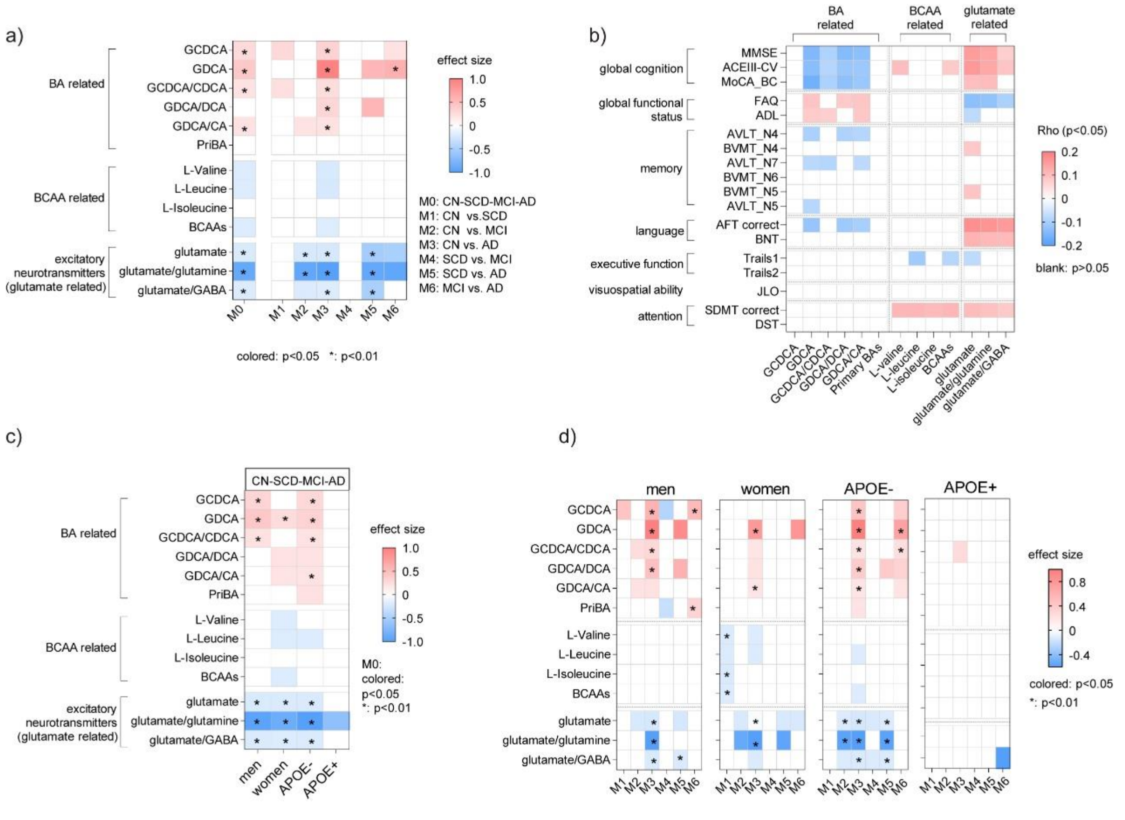

Figure S3. Performances of the 13 identified features in age-matched sub-populations.

a) Effect sizes of the features among four clinical stages (M0) and between every two stages (M1-M6) based on linear regression models (M0) and logistic regression models (M1-M6) respectively. Colored cell indicates p<0.05 and * indicates p<0.01 (two-sided. b) Associations of the features and cognition scores. Cell color indicates correlation coefficient from Partial Spearman analysis (two-sided). c) Effect sizes of the features when differentiating four clinical stages based on linear regression models (M0) in sex and *APOE*-ε4 status stratified populations. Colored cell indicates p<0.05 and * indicates p<0.01 (two-sided). d) Effect sizes of the features when differentiating every two stages in sex and *APOE*-ε4 stratified populations. Colored cell indicates p<0.05 and * indicates p<0.01 (two-sided). Covariates including age, sex, BMI, education year, and *APOE*-ε4 were adjusted in all the above analyses when applicable.

GCDCA: Chenodeoxycholic acid glycine conjugate; GDCA: Deoxycholic acid glycine conjugate; GCDCA/CDCA: the ratio of Chenodeoxycholic acid glycine conjugate and Chenodeoxycholic acid; GDCA/DCA: the ratio of Deoxycholic acid glycine conjugate and Deoxycholic acid; GDCA/CA: the ratio of Deoxycholic acid glycine conjugate and Cholic acid; PriBA: concentration summation of primary BAs (CA+CDCA+GCA+TCA+GCDCA+TCDCA); MMSE: Mini-Mental State Examination; ACEIII-CV: Chinese version of Addenbrooke's cognitive examination-III; MoCA-BC: Chinese version of Montreal Cognitive Assessment-Basic; FAQ: Functional Assessment Questionnaire; ADL: Activities of Daily Living; AVLT: Auditory Verbal Learning Test; BVMT-R: Brief Visuospatial Memory Test-Revised; N4: short delayed recall; N5:

long delayed recall; N6/N7: recognition; AFT: Animal Verbal Fluency Test; BNT: Boston Naming Test; STT-A and B: Shape Trail Test Part A and B; JLO: Judgement of Line Orientation; SDMT: Symbol Digit Modalities Test; DST: Digit Span Test.

Table S4. Metabolite measurement information of 8 validation data sets.

Data set name	Testing platform or assay	Testing team	qualitative / quantitative	ADNI phase
ADNI-Duke2016	The AbsoluteIDQ p180 assay	Duke University	quant	ADNI1
ADNI-Duke2017	The AbsoluteIDQ p180 assay	Duke University	quant	ADNI2, GO
ADNI-California2017	Gas chromatography time of flight mass spectrometry (GC-TOF/MS) instrument	University of California	qual	ADNI1
ADNI-Hawaii2021	Ultra-performance liquid chromatography coupled to tandem mass spectrometry (UPLC-MS/MS).	University of Hawaii Cancer Center	quant	ADNI1, GO, 2
ADNI-Nightingale2021	Nuclear Magnetic Resonance (NMR)	Nightingale Health's NMR metabolomics platform	quant	ADNI1, GO, 2
ADNI-DukeBAs2016	The Biocrates Bile Acids assay	Duke University	quant	ADNI1
ADNI-M2OVEAD2016	NA	NA	qual	NA
Rosmap-Hawaii2017	Gas chromatography time of flight mass spectrometry (GC-TOF/MS) instrument	University of Hawaii Cancer Center	qual	NA

Table S5. Population characteristics of 8 validation data sets.

ADNI-Duke2016 (serum)	All (n=818)	CN (n=232)	LMCI (n=397)	AD (n=189)	
Age(yr)	75.23+6.83	75.89+5.06	74.78+7.45#	75.37+7.29	
Sex(M/F)	469/349*	119/113	257/140#	93/96#	
Education(yr)	15.53+3.02*	16.06+2.84	15.62+3.02	14.67+3.09#	
APOE(ε4+)%	37.53%*	24.14%	40.81%#	47.09%#	
ADAS13	18.48+9.29*	9.4+4.21	18.76+6.24#	29.02+7.56#	
ADNI-Duke2017 (serum)	All (n=898)	CN (n=182)	SMC (n=104)	EMCI/LMCI (n=474)	AD (n=138)
Age(yr)	72.46+7.28*	73.45+6.32	72.18+5.55	71.54+7.49#	74.55+8.3
Sex(M/F)	472/426*	88/94	45/59#	256/218#	83/55#
Education(yr)	16.27+2.62*	16.6+2.53	16.82+2.55	16.18+2.6#	15.75+2.77#
APOE(ε4+)%	35.75%*	24.73%	31.73%	37.76%#	46.38%#
ADAS13	15.42+9.65*	9.07+4.5	8.69+4.09	14.76+6.8#	31.12+8.54#
ADNI-California2017 (serum)	All (n=820)	CN (n=232)	LMCI (n=398)	AD (n=190)	
Age(yr)	75.23+6.82	75.89+5.06	74.79+7.44#	75.35+7.28	
Sex(M/F)	470/350*	119/113	258/140#	93/97#	
Education(yr)	15.53+3.02*	16.06+2.84	15.62+3.01	14.68+3.09#	
APOE(ε4+)%	37.44%*	24.14%	40.70%#	46.84%#	
ADAS13	18.46+9.28*	9.4+4.21	18.74+6.25#	28.97+7.58#	
ADNI-Hawaii2021 (serum)	All (n=1172)	AD(n=186)	CN(n=350)	EMCI/LMCI (n=636)	
Age(yr)	73.90+7.11*	75.30+7.70	74.82+5.71	72.98+7.49#	
Sex(M/F)	649/523*	101/85#	181/169	367/269#	
Education(yr)	15.94+2.84*	14.98+2.99#	16.36+2.74	15.99+2.79#	
APOE(ε4+)%	36.95%*	25.43%#	39.62%	49.46%#	
ADAS13	16.26+8.99*	28.97+7.76#	9.11+4.21	16.48+6.78#	
ADNI-Nightingale Health2021 (serum)	All (n=1681)	CN (n=404)	SMC (n=104)	EMCI/LMCI (n=854)	n=AD (n=319)
Age(yr)	73.78+7.21*	74.78+5.81	72.18+5.55#	73.05+7.63#	75.30+7.70
Sex(M/F)	917/764*	202/202	45/59#	500/354#	170/149#
Education(yr)	15.93+2.83*	16.31+2.72	16.82+2.55	15.93+2.78#	14.98+2.99#
APOE(ε4+)%	36.59%*	24.26%	31.73%	39.23%#	46.71%#
ADAS13	16.82+9.62*	9.16+4.30	8.69+4.09	16.53+6.86#	28.98+7.76#
ADNI-DukeBAs2016 (serum)	All (n=833)	AD (n=191)	CN (n=233)	EMCI/LMCI (n=399)	
Age(yr)	74.35+10.64*	75.36+7.26	75.94+5.11	74.80+7.44#	
Sex(M/F)	473/360*	94/97#	120/113	259/140#	
Education(yr)	15.35+3.45*	28.97+7.56#	9.43+4.23	15.63+3.02	
APOE(ε4+)%	36.97%*	47.12%#	24.03%	40.60%#	

ADAS13	18.25+9.43*	28.97+7.56#	9.43+4.23	18.73+6.25#	
ADNI-M2OVEAD2016 (serum)	All (n=897)	AD (n=138)	CN (n=182)	EMCI/LMCI (n=473)	SMC (n=104)
Age(yr)	72.47+7.28*	74.55+8.30	73.45+6.32	71.55+7.49#	72.18+5.55
Sex(M/F)	472/425*	83/55#	88/94	256/217#	45/59#
Education(yr)	16.27+2.62*	15.75+2.77#	16.60+2.53	16.18+2.61	16.82+2.55
APOE(ε4+)%	35.67%*	46.38%#	24.73%	37.63%#	31.73%
ADAS13	15.42+9.66*	31.12+8.54#	9.07+4.50	14.767+6.806#	8.69+4.09
Rosmap-Hawaii2017 (serum)	All (n=566)	AD (n=13)	CN (n=446)	MCI (n=107)	
Age(yr)	82.28+7.49*	86.12+8.88#	81.13+7.38	86.65+6.02#	
Sex(M/F)	447/119	10/3#	356/90	81/26	
BMI	27.61+5.48*	24.80+6.71	28.01+5.50	26.23+4.95#	
Education(yr)	15.68+3.05*	17.15+3.76	15.71+3.06	15.26+2.85	
Global cognition	0.19+0.59	-1.23+0.77	0.38+0.44	-0.41+0.48	
APOE(ε4+)%	17.49%*	30.77%	16.14%	22.12%	

Data are presented as mean+S.D., percentage, or number. * indicates Chi-squared test, analysis of variance, or Kruskal–Wallis test FDR<0.05 comparing 4 groups (adjusted by Benjamini and Hochberg). # indicates Chi-squared test, student's t-test or Mann-Whitney test FDR<0.05 compared to CN (adjusted by Benjamini and Hochberg). CN: cognitively normal; AD: Alzheimer's disease; SCD: subjective cognitive decline; MCI: mild cognitive impairment; EMCI: early MCI; LMCI: late MCI; SMC: significant memory concern.

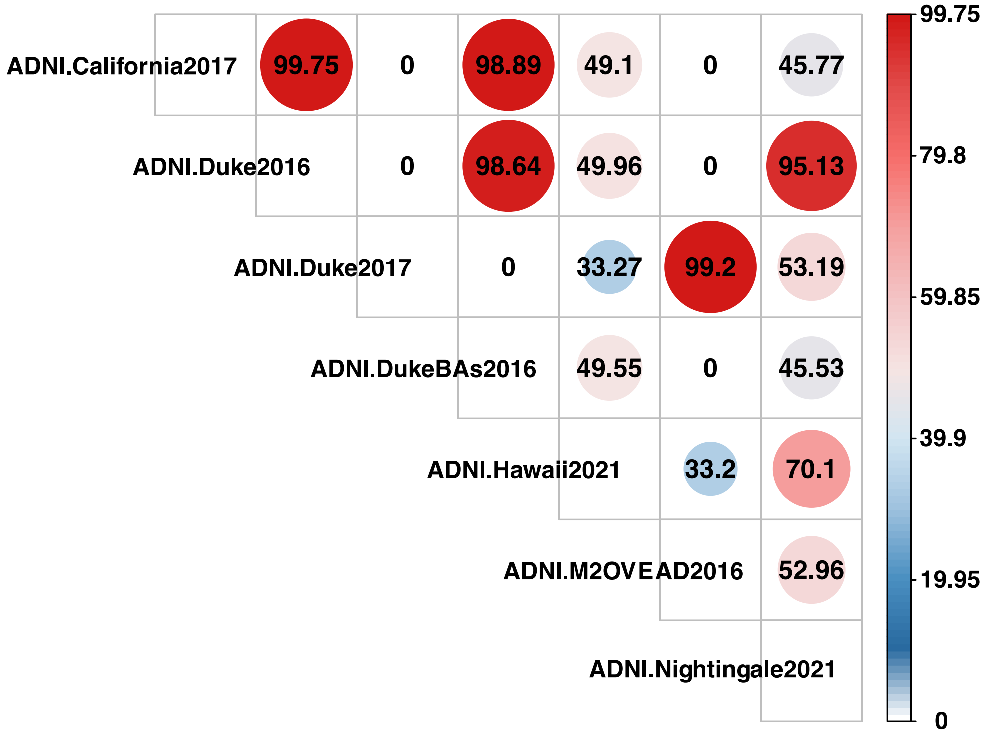

Figure S4 The percentage of overlapping patients with respect to the overall number of unique subjects between both studies (i.e Intersection over Union).

Figure S5. The meta-analysis forest plots of identified features on their standardized mean differences of CN and AD.

GCDCA

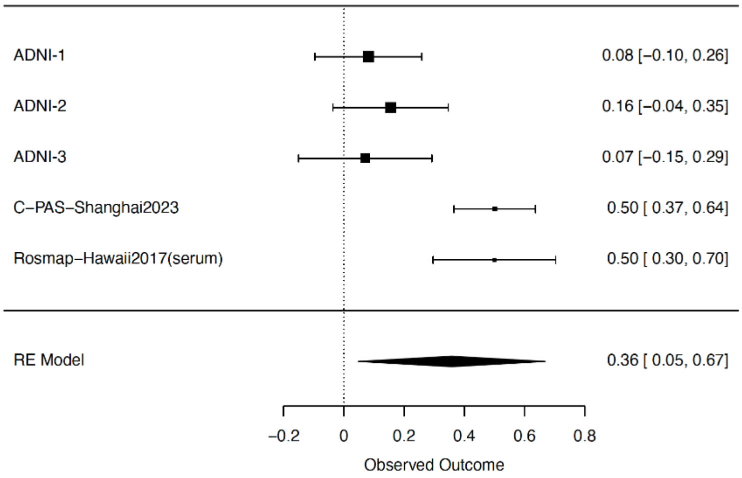

GDCA

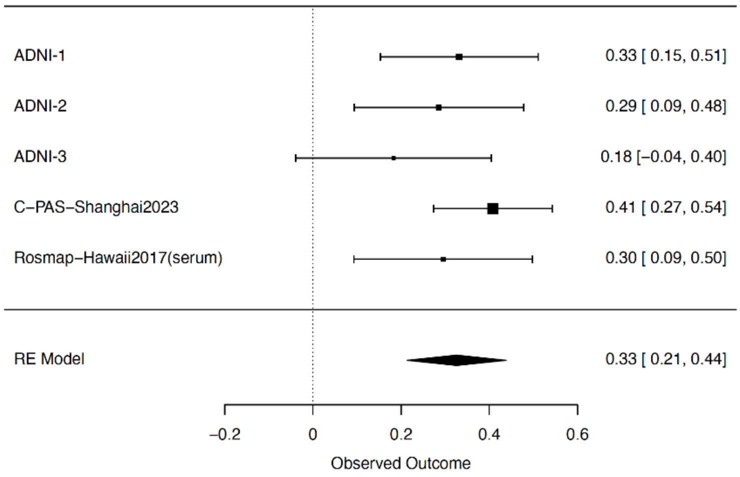

GCDCA/CDCA

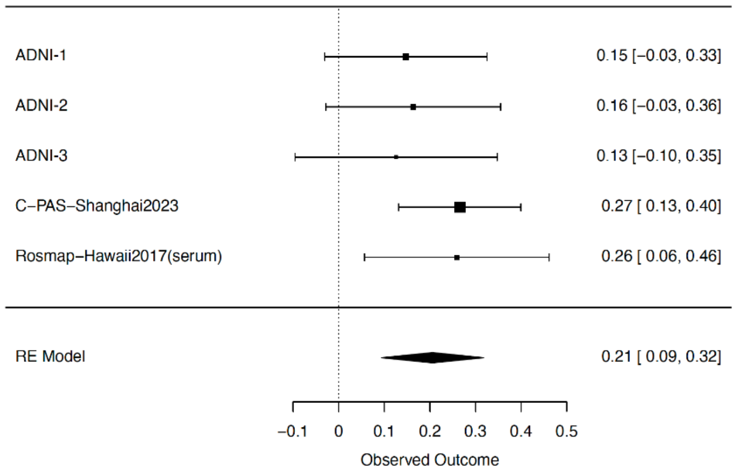

GDCA/DCA

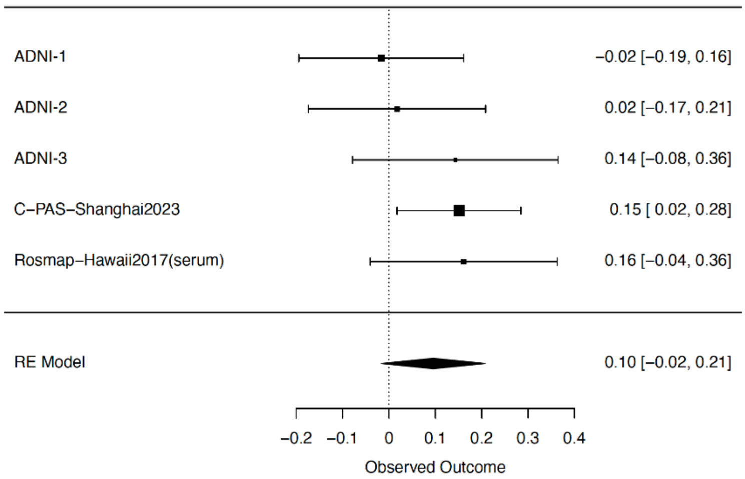

GDCA/CA

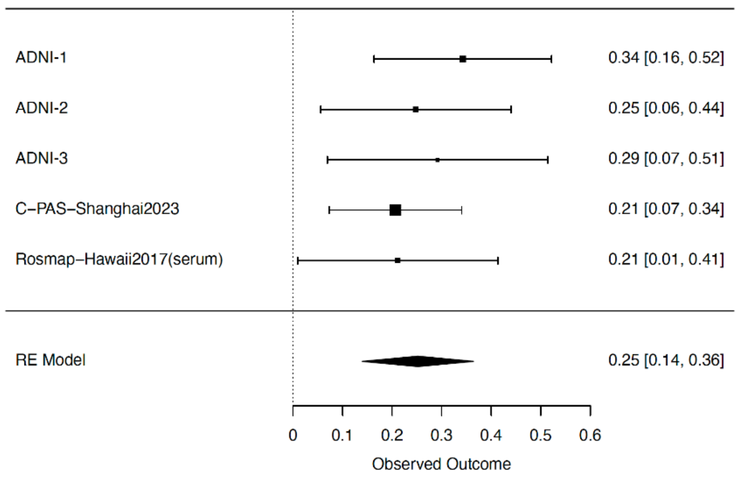

PriBA

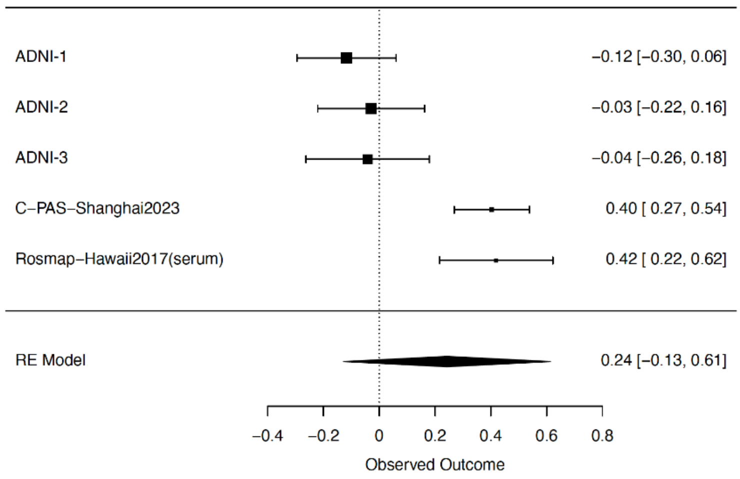

Valine

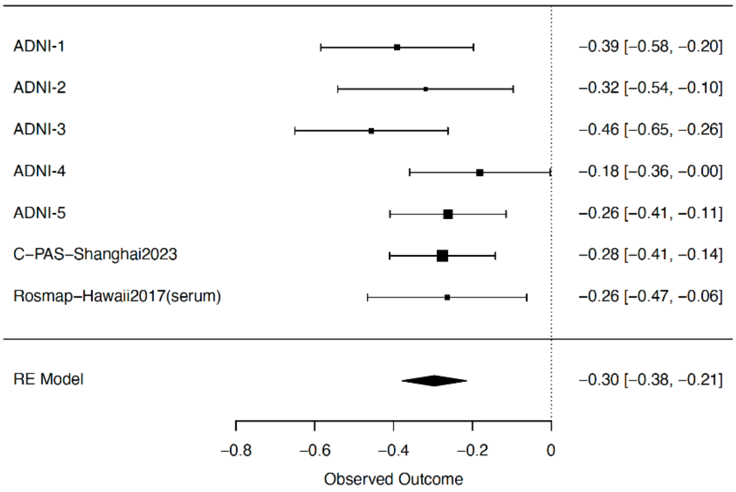

Leucine

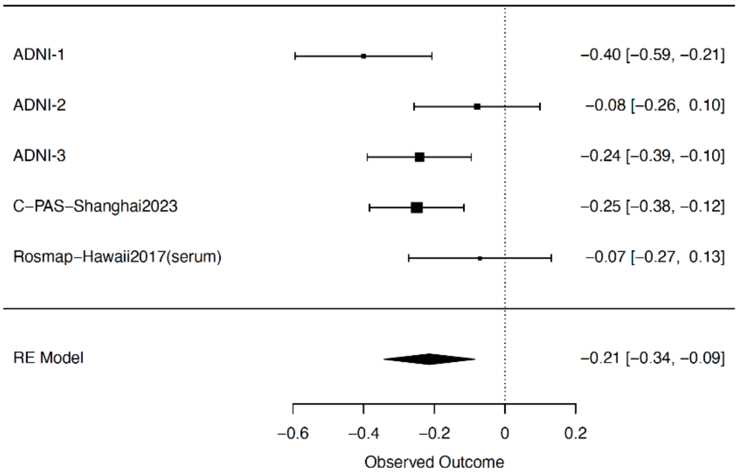

Isoleucine

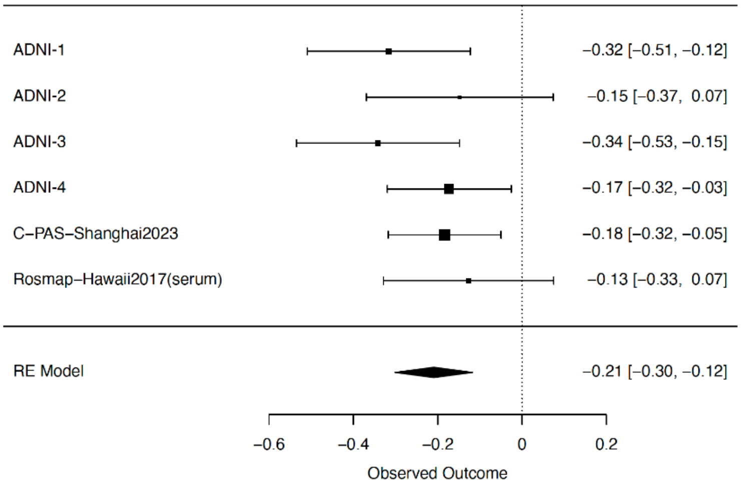

BCAAs

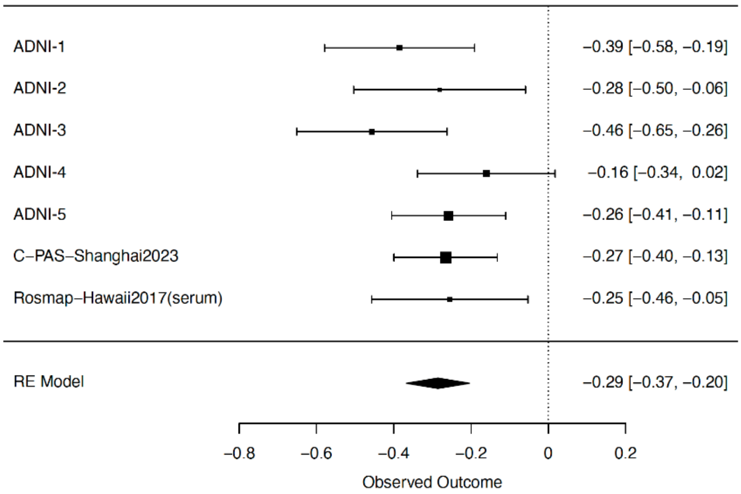

Glutamate

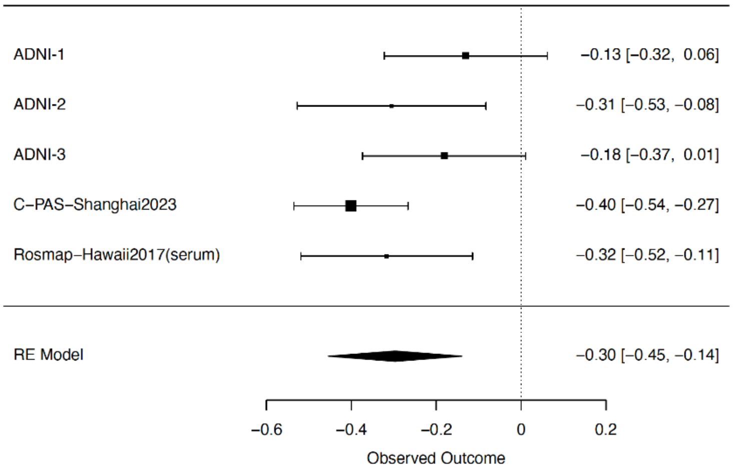

Glutamate/Glutamine

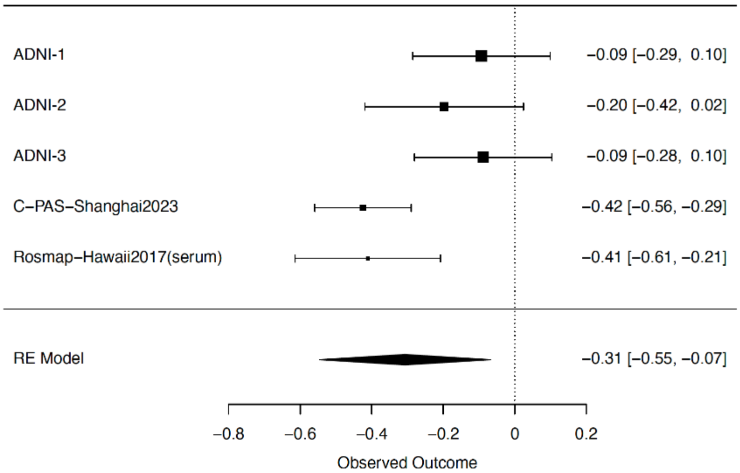

Glutamate/GABA

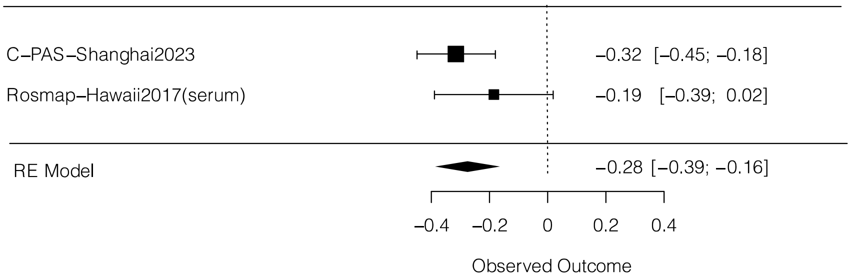

Figure S6. The meta-analysis forest plots of identified features on their Partial Spearman correlation coefficients with global cognition.

GCDCA

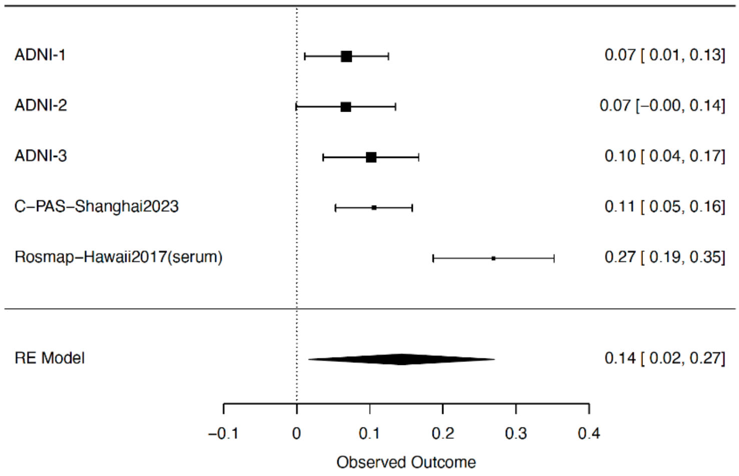

GDCA

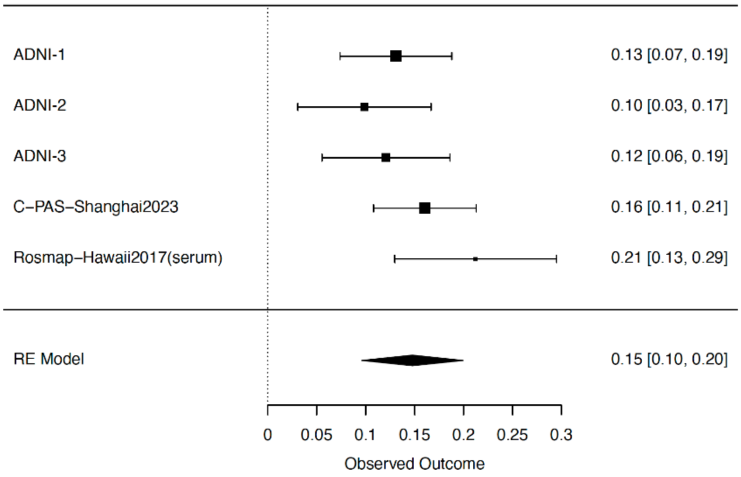

GCDCA/CDCA

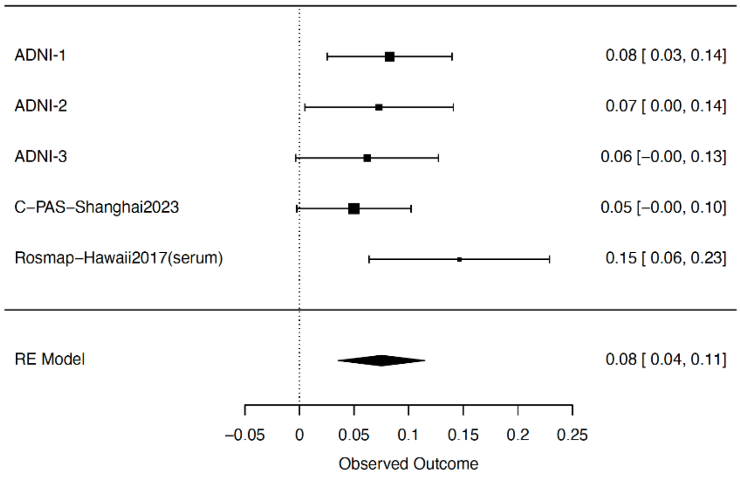

GDCA/DCA

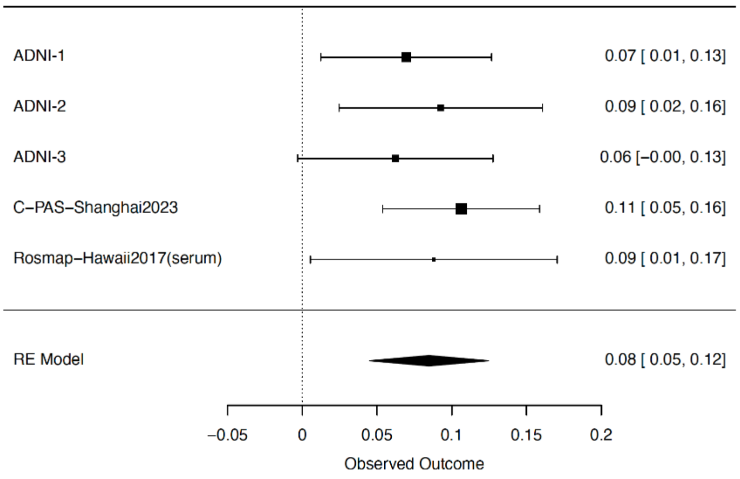

GDCA/CA

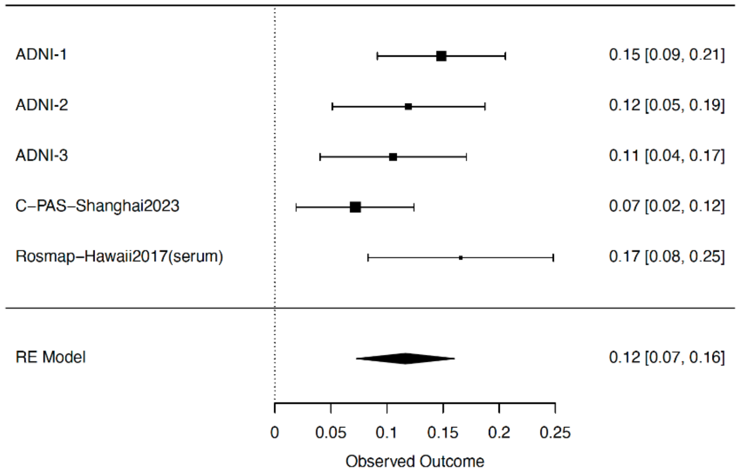

PriBA

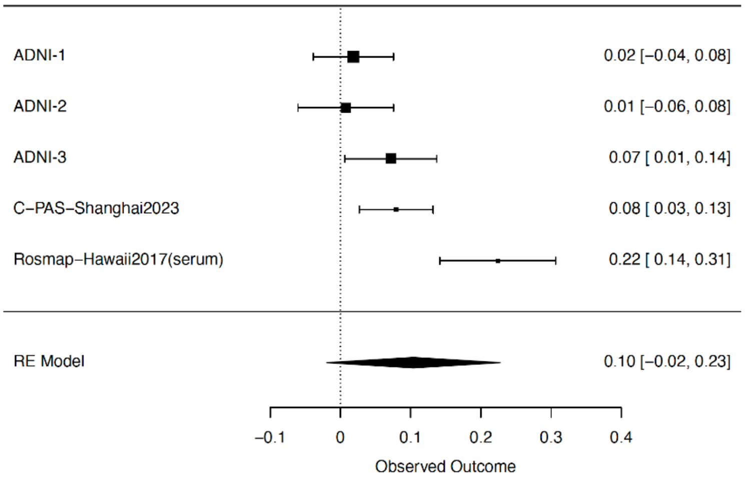

Valine

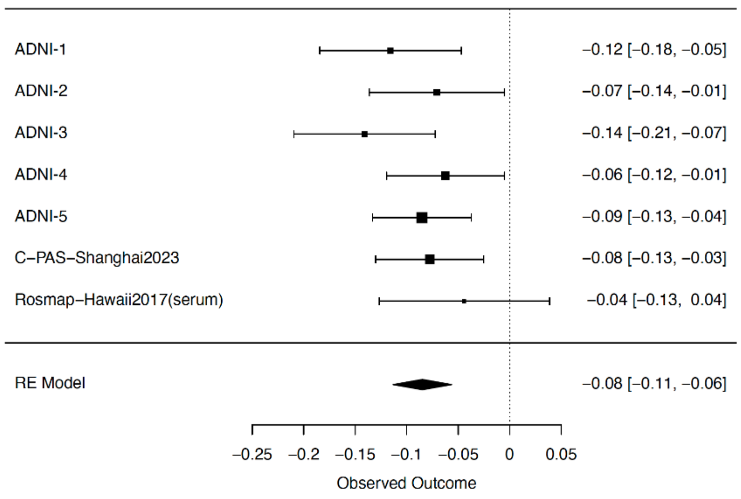

Leucine

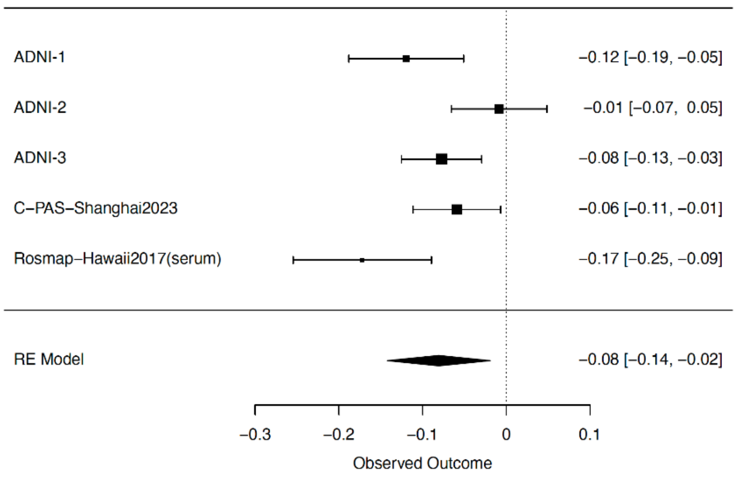

Isoleucine

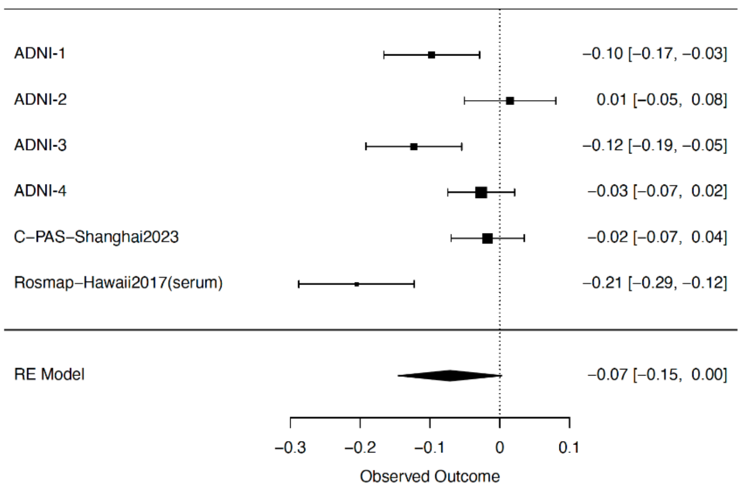

BCAAs

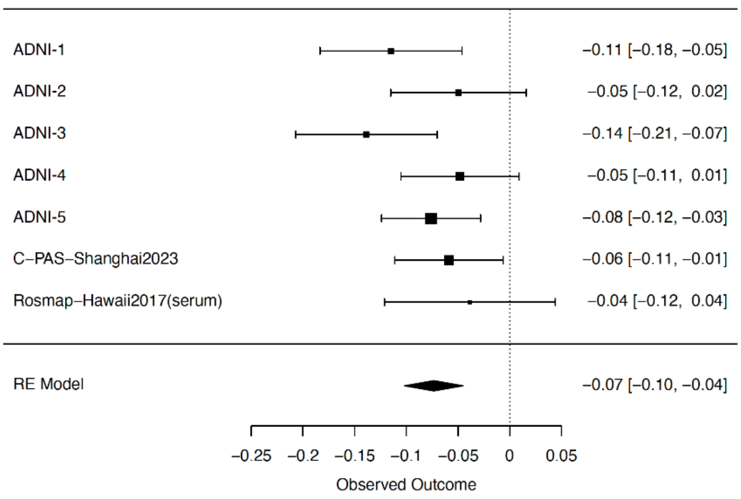

Glutamate

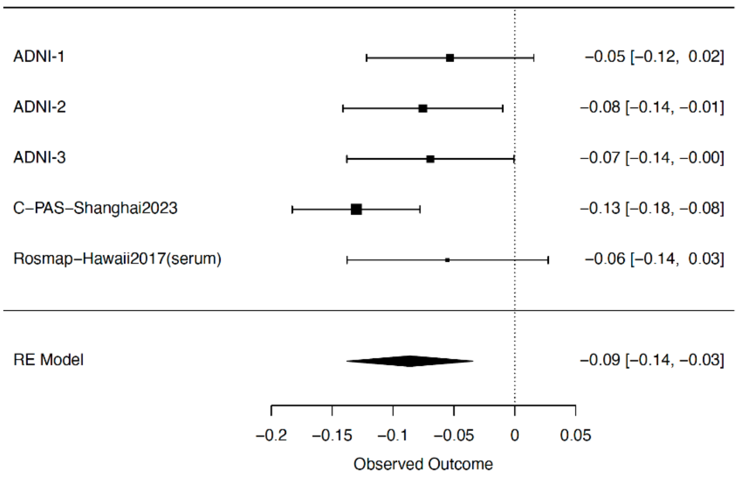

Glutamate/Glutamine

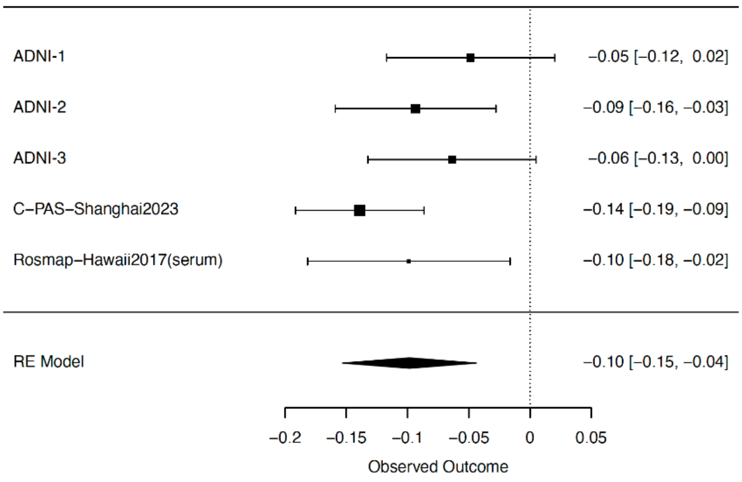

Glutamate/GABA

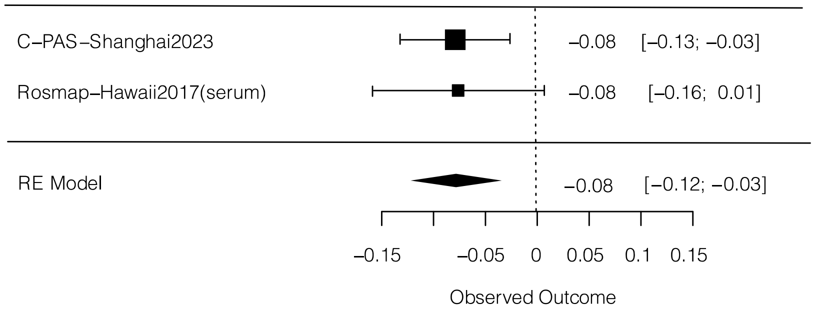

Cognition scores for ADNI, C-PAS, and ROSMAP data sets were ADAS-13, -1*MMSE, and a composite measure of global cognition created by averaging the z-scores of all tests respectively.

Methods

1. Neuropsychological measurements and clinical diagnosis in C-PAS

All participants in the C-PAS study had to fulfill the following criteria: (1) Age between 40 and 90 years with a minimum education duration of more than 1 year; (2) Absence of severe hearing or visual impairment, and proficiency in Mandarin communication; (3) Willingness to complete neuropsychological tests, cranial MRI, brain PET scans, and blood biomarker assessments; (4) No history of stroke, craniocerebral injury, brain tumor, anxiety, depression, or other conditions potentially impacting cognitive function adversely.

Brief cognitive screening tests included the Chinese version of Mini-Mental State Examination (MMSE)[1], Montreal Cognitive Assessment-Basic (MoCA-BC)[2] and Addenbrooke's Cognitive Examination-III (ACE-III-CV)[3]. Various cognitive domains were evaluated through standardized neuropsychological tests: Auditory Verbal Learning Test (AVLT)[4] and Brief Visuospatial Memory Test-Revised (BVMT-R)[5] for memory; Boston Naming Test (BNT)[6] and Animal Verbal Fluency Test (AFT)[7] for language; Shape Trail Test Part A and B (STT-A, STT-B)[8] for executive function; Judgement of Line Orientation(JLO)[9] for visuospatial ability; Symbol Digit Modalities Test (SDMT) and Digit Span Test(DST)[10] for attention. Global functional status was assessed by Activities of Daily Living (ADL)[11] and Functional Assessment Questionnaire (FAQ)[12].

Participants with no cognitive complaint and objective cognitive impairment assessed via neuropsychological tests were defined as cognitively normal (CN). Those with self-reported memory decline but performed essentially normal on neuropsychological tests were classified as Subjective Cognitive Decline (SCD) according to the conceptual framework proposed by the working group of SCD Initiative (SCD-I)[13]. Mild cognitive impairment (MCI) was diagnosed according to the actuarial neuropsychological criteria put forward by Jak and Bondi[14]. Participants were classified as having dementia according to the criteria of Diagnostic and Statistical Manual of Mental Disorders, 4th edition- revised, and the clinical diagnosis of probable AD dementia was made according to the NIA-AA criteria[15].

2. Brain PET neuroimaging acquisition and preprocessing

[18F]Florbetapir PET/CT scans were employed to evaluate Aβ plaques in the brain. The tracer was produced in the Department of Nuclear Medicine & PET Center, Huashan Hospital, Fudan University, adhering to Good Manufacturing Practice (GMP) conditions.

PET/CT imaging was performed using PET/CT scanners (Biograph mCT Flow, Siemens, Erlangen, Germany) with parameters described previously[16, 17]. Twenty-minute scans were conducted 50 minutes post-injection of approximately ~37 MBq/kg (±10%) of [18F]florbetapir intravenously. Following acquisition, the PET images underwent reconstruction using a filtered back-projection algorithm with corrections for decay, normalization, dead time, photon attenuation, scatter, and random coincidences. PET image preprocessing was conducted using SPM12 (Welcome Trust Centre for Neuroimaging, London, UK; https://www.fil.ion.ucl.ac.uk/spm) and CAT12 (http://www.neuro.uni-jena.de/cat) following a previously outlined procedure[18, 19]. After reorienting PET and T1-weighted MR images, PET images were co-registered to individual T1-weighted images. Subsequently, the T1-weighted images were segmented using CAT12, and the generated tissue-labeled images were utilized for partial volume correction (PVC) of PET images employing the Muller-Gartner method[20]. Then the deformation field file from segmentation was used to transform corresponding PET images into the MNI space, and finally images were smoothed using a Gaussian filter with a full width at half of the maximum (FWHM) equal to 8 mm. The global cortical Aβ burden was computed using the preprocessed images in MNI space, represented as the mean SUVr in cortical area, including posterior cingulate, precuneus, frontal, lateral parietal, lateral temporal, medial temporal, and occipital regions.

3. Quantitative measurement and pretreatment of metabolic data (C-PAS)

Targeted metabolites (n=189) from 12 metabolite types were quantitatively measured using a metabolite array technology (developed by our group in 2021) with an ultra-performance liquid chromatography coupled to tandem mass

spectrometry system (UPLC-MS/MS) [21]. This automatic and high-throughput system allows for the simultaneous determination of as many metabolites as possible, approximately 200, spanning various classes. We constructed a combined MS library of 3-NPH derivatives from structurally diverse compounds to facilitate metabolite identification. The system demonstrated excellent linearity, reproducibility, and stability, making it suitable for large clinical applications. However, it's important to note that, like any technology, there are limitations. The coverage of certain metabolite classes, such as many lipid classes, is limited to ensure stability and accuracy.

All the samples were prepared and quantified by the same staff using the same protocol. Lab staffs were blinded to diagnosis and pathological data. In addition to the internal standards for quality control, test mixtures (a group of commercially available standards with a mass range across the system mass range at 3 concentrations, low, medium, and high, within the range of the calibration curves), and pooled biological samples were used as well. The quality control samples were evenly inserted in the running sequence to monitor the stability of the analysis. The raw data files were processed using the TMBQ software (V1.0, HMI, Shenzhen, China) including peak integration, calibration, quantification, quality control, and batch effect adjustment for each metabolite, according to the manufacturer's instructions. In total, 199 metabolites were measured, and 10 that fell below the limit of quantification were excluded from subsequent analysis. Outliers were identified using Cauchy distribution robust fit (K sigma=7). Outliers (<0.2%) and missing values (<0.1%) were replaced using multivariate normal imputation. The data were logarithmic transformed (base=2) to normalize their distribution for statistical analysis.

4. Meta-analysis

A total of 9 data sets were involved in the meta-analysis, including 7 from ADNI, 1 from ROSMAP, and our C-PAS. These data sets had to meet the following criteria: 1) derived from peripheral blood samples; 2) from AD-related studies with clinical or pathological diagnosis (containing at least CN and AD stages); 3) with at least one

indicator of global cognitive function; 4) with age and sex information; 5) with test results for at least one of the 13 metabolic features; 6) related data are accessible.

Statistical independence constitutes a fundamental assumption in a meta-analysis when pooling effect sizes [22]. The presence of dependency between samples or data can artificially attenuate heterogeneity, potentially leading to spurious positive findings [23]. This issue is known as the unit-of-analysis error. Considering the overlaps of samples in ADNI studies, we employed the three-level meta-analysis models on the identified features to evaluate their standardized mean differences between CN and AD ((mean level of ADs - mean level of CNs) / S.D. of CNs and ADs) and their correlations with global cognition (partial spearman correlation adjusting age and sex). Features were scaled to 0-1 within each data set to correct batch effect. Outliers (<0.2%) were identified by Local Outlier Factor and were excluded for analysis. All applicable data sets and the independence between them (which datasets have duplicate samples) were entered at once.

The three-level meta-analysis model is a meta-analysis method specifically designed to address dependencies between samples or data [24, 25]. A three-level model consists of three levels of pooling. Initially, researchers combine the results of individual participants within their primary studies to report an aggregated effect size / correlation. Subsequently, at level 2, these effect sizes / correlations are nested within multiple clusters. Finally, pooling the aggregated cluster effects / correlations yields the overall true effect size μ.

Level 1 model:

$$\hat{\theta}_{ij} = \theta_{ij} + \epsilon_{ij}$$

Level 2 model:

$$\theta_{ij} = \kappa_j + \varsigma_{(2)ij}$$

Level 3 model:

$$\kappa_j = \mu + \varsigma_{(3)j}$$

Here, $\hat{\theta}_{ij}$ represents an estimate of the true effect size θ_{ij}, with ij indicating "effect size i nested in cluster *j*." The parameter κ_j denotes the average effect size within cluster *j*,

while μ represents the overall average population effect. These formulas can be combined into a single line as follows:

$$\hat{\theta}ij = \mu + \varsigma(2)_{ij} + \varsigma(3)_j + \epsilon_{ij}$$

This formula now encompasses two sources of heterogeneity: $\varsigma(2)_{ij}$, signifying within-cluster heterogeneity at level 2, and $\varsigma(3)_j$, representing between-cluster heterogeneity at level 3. Consequently, fitting a three-level meta-analysis model necessitates estimating not only one heterogeneity variance parameter ($\tau 2$) but also two (one for level 2 and another for level 3).

We fitted the three-level meta-analysis models using the rma.mv function in the metafor package, employing maximum likelihood procedures[26].

Discussion

1. Potential Impacts of Age, Sex, Education Year, and *APOE*-ε4 Status on the Association of Metabolic Profiles and AD Progression

The association of metabolic profiles with AD progression is intricately influenced by various factors. Factors such as age, sex, education year, and *APOE*-ε4 status are recognized as significant contributors, often exerting greater impacts than other variables[27, 28, 29].

Age: Age, a primary non-modifiable risk factor for AD, is intricately linked to alterations in metabolic profiles during cognitive aging and AD development. These changes encompass dysregulated levels of BCAAs, modified bile acids, abnormal glutamine-glutamate cycle, impaired beta-amyloid clearance, and alterations in phosphatidylcholines (PCs) and sphingomyelins (SMs) in fatty acid composition and levels[30]. Specific metabolites exhibit differential associations with cognitive decline and AD across various age groups. For instance, serum total cholesterol (TC) and low-density lipoprotein cholesterol (LDL-C) represent age-dependent risk factors for cognitive impairment among elderly participants, suggesting potential interactions between metabolites and age in AD[28]. Metabolome-wide association studies support this, revealing several metabolite-by-age interactions significantly correlated with executive function, an early aspect of cognition affected during AD progression[29]. The differential rates of metabolism for these particular metabolites could explain age-specific associations, necessitating compensatory mechanisms during younger years to account for rapid metabolism[29].

APOE-ε4 Status: The influence of *APOE*-ε4 as a risk factor for AD has long been recognized, impacting cholesterol transport, brain lipid composition, beta-amyloid elimination, and neuroinflammation[31]. Recently, it has emerged as a modifier of AD metabolism, with stratified analyses demonstrating *APOE*-ε4-dependent heterogeneous effects of metabolites on AD. Certain metabolites, such as PCs and proline, exhibit specific effects in female ε4 carriers[27].

Sex: Sex-based modulation of the associations between metabolites and AD biomarkers has been substantiated, with direct evidence pointing to sex-dependent

alterations in various metabolic pathways, including GABA synthesis, arginine biosynthesis, alanine, aspartate, and glutamate metabolism, fatty acid elongation, and lysophospholipid metabolism[32]. Stratified analysis on ADNI data has highlighted substantial heterogeneity between sexes, emphasizing potential sex-specific interactions of metabolites and dysregulations in energy metabolism, energy homeostasis, and stress response. Notably, specific metabolic effects were identified in female ε4 carriers[27, 28].

Education: Education is linked to enhanced cognitive reserve and improved metabolic performance associated with AD, yet its potential influence on the association between metabolites and AD remains ambiguous[31]. A Mendelian randomization study confirmed that the protective effect of education against AD is largely attributable to better cognition[33]. Individuals with higher education tend to participate in brain-stimulating activities and adopt healthier lifestyles, which are conducive to metabolic health[34].

Multiple factors: Studies addressing the interplay of multiple factors on the association between metabolic profiles and AD are in their early stages. Distinct alterations in fatty acid metabolomics have been observed in *APOE*-ε4 non-carriers and women, suggesting a nuanced role for *APOE*-ε4-sex intertwined effects in metabolic pathways relevant to AD[35]. Age-related decreases in glutamate, GABA, and sphingolipids worsened with the increase of *APOE*-ε4 load, potentially contributing to deficits in synaptic, learning, and memory-related functions[36]. Interactions between sex and age have been underscored, supported by sex- and age-tailored correlations between serum lipids and cognitive impairment[28].

References

1. Katzman R*, et al.* A Chinese version of the Mini-Mental State Examination; impact of illiteracy in a Shanghai dementia survey. *J Clin Epidemiol* **41**, 971-978 (1988).

2. Huang L*, et al.* Chinese version of Montreal Cognitive Assessment Basic for discrimination among different severities of Alzheimer's disease. *Neuropsychiatr Dis Treat* **14**, 2133-2140 (2018).

3. Pan FF, Wang Y, Huang L, Huang Y, Guo QH. Validation of the Chinese version of Addenbrooke's cognitive examination III for detecting mild cognitive impairment. *Aging Ment Health* **26**, 384-391 (2022).

4. Zhao Q*, et al.* Auditory Verbal Learning Test is Superior to Rey-Osterrieth Complex Figure Memory for Predicting Mild Cognitive Impairment to Alzheimer's Disease. *Curr Alzheimer Res* **12**, 520-526 (2015).

5. Benedict RHB, Schretlen, D., Groninger, L., Dobraski, M., & Shpritz, B. . Revision of the Brief Visuospatial Memory Test: Studies of normal performance, reliability, and validity. . *Psychological Assessment* **8**, 145–153 (1996).

6. Guo Q.H. HZ, Shi W.X., Sun Y.M., Lv C.Z. Boston naming test using by Chinese elderly, patient with mild cognitive impairment and Alzheimer's dementia. *Journal of Chinese Mental Health* **20**, 81-85 (2006).

7. Zhao Q, Guo Q, Hong Z. Clustering and switching during a semantic verbal fluency test contribute to differential diagnosis of cognitive impairment. *Neurosci Bull* **29**, 75-82 (2013).

8. Zhao Q, Guo Q, Li F, Zhou Y, Wang B, Hong Z. The Shape Trail Test: application of a new variant of the Trail making test. *PLoS One* **8**, e57333 (2013).

9. Qualls CE, Bliwise NG, Stringer AY. Short forms of the Benton Judgment of Line Orientation Test: development and psychometric properties. *Arch Clin Neuropsychol* **15**, 159-163 (2000).

10. Wechsler D. Wechsler Adult Intelligence Scale—Fourth edition: Technical and interpretive manual. (2008).

11. Chen P, Yu ES, Zhang M, Liu WT, Hill R, Katzman R. ADL dependence and medical conditions in Chinese older persons: a population-based survey in Shanghai, China. *J Am Geriatr Soc* **43**, 378-383 (1995).

12. Pfeffer RI, Kurosaki TT, Harrah CH, Jr., Chance JM, Filos S. Measurement of functional activities in older adults in the community. *J Gerontol* **37**, 323-329 (1982).

13. Huang L, Chen K, Liu Z, Guo Q. A Conceptual Framework for Research on Cognitive Impairment with no Dementia in Memory Clinic. *Curr Alzheimer Res* **17**, 517-525 (2020).

14. Bondi MW*, et al.* Neuropsychological criteria for mild cognitive impairment improves diagnostic precision, biomarker associations, and progression rates. *J Alzheimers Dis* **42**, 275-289 (2014).

15. McKhann GM*, et al.* The diagnosis of dementia due to Alzheimer's disease: recommendations from the National Institute on Aging-Alzheimer's Association workgroups on diagnostic guidelines for Alzheimer's disease. *Alzheimer's & dementia : the journal of the Alzheimer's Association* **7**, 263-269 (2011).

16. Zhao Z*, et al.* 18F-AV45 PET and MRI Reveal the Influencing Factors of Alzheimer's Disease Biomarkers in Subjective Cognitive Decline Population. *Journal of Alzheimers Disease* **93**, (2023).

17. Wang J*, et al.* The Effect of Gender and APOE ε4 Status on Brain Amyloid-β Deposition in Different Age Groups of Mild Cognitively Impaired Individuals: A PET-CT Study. *Journal of Alzheimers Disease* **94**, 763-775 (2023).

18. Huang Q*, et al.* Changes in brain glucose metabolism and connectivity in somatoform disorders: an 18F-FDG PET study. *European Archives of Psychiatry and Clinical Neuroscience* **270**, 881-891 (2020).

19. Gasera C, Dahnkea R, Thompsonb PM, Kurth F, Luders E. CAT – A Computational Anatomy Toolbox for the Analysis of Structural MRI Data. *bioRxiv*, (2022).

20. Gonzalez-Escamilla G, Lange C, Teipel S, Buchert R, Grothe MJ, Initiative ftAsDN. PETPVE12: an SPM toolbox for Partial Volume Effects correction in brain PET – Application to amyloid imaging with AV45-PET. *NeuroImage* **147**, 669-677 (2017).

21. Xie G*, et al.* A Metabolite Array Technology for Precision Medicine. *Anal Chem* **93**, 5709-5717 (2021).

22. Higgins JP, Green S. Cochrane handbook for systematic reviews of interventions. (2008).

23. Borenstein M, Hedges LV, Higgins JP, Rothstein HR. *Introduction to meta-analysis*. John Wiley & Sons (2021).

24. Cheung MW-L. Modeling dependent effect sizes with three-level meta-analyses: a structural equation modeling approach. *Psychological methods* **19**, 211 (2014).

25. Assink M, Wibbelink CJ. Fitting three-level meta-analytic models in R: A step-by-step

tutorial. *The Quantitative Methods for Psychology* **12**, 154-174 (2016).

26. Harrer M, Cuijpers P, Furukawa TA, Ebert DD. *Doing meta-analysis with R: A hands-on guide*. CRC press (2021).

27. Arnold M, *et al.* Sex and APOE ε4 genotype modify the Alzheimer's disease serum metabolome. *Nature communications* **11**, 1148 (2020).

28. Zhao B, *et al.* The gender- and age- dependent relationships between serum lipids and cognitive impairment: a cross-sectional study in a rural area of Xi'an, China. *Lipids Health Dis* **18**, 4 (2019).

29. Darst BF, *et al.* Metabolites Associated with Early Cognitive Changes Implicated in Alzheimer's Disease. *Journal of Alzheimer's Disease : JAD* **79**, 1041-1054 (2021).

30. Yu Z, *et al.* Human serum metabolic profiles are age dependent. *Aging Cell* **11**, 960-967 (2012).

31. 2021 Alzheimer's disease facts and figures. *Alzheimers Dement* **17**, 327-406 (2021).

32. Strefeler A, *et al.* Molecular insights into sex-specific metabolic alterations in Alzheimer's mouse brain using multi-omics approach. *Alzheimer's Research & Therapy* **15**, 8 (2023).

33. Lord J, *et al.* Disentangling Independent and Mediated Causal Relationships Between Blood Metabolites, Cognitive Factors, and Alzheimer's Disease. *Biol Psychiatry Glob Open Sci* **2**, 167-179 (2022).

34. Green R, *et al.* Metabolic correlates of late midlife cognitive outcomes: findings from the 1946 British Birth Cohort. *Brain Commun* **4**, fcab291 (2022).

35. González-Domínguez R, *et al.* Apolipoprotein E and sex modulate fatty acid metabolism in a prospective observational study of cognitive decline. *Alzheimer's Research & Therapy* **14**, 1 (2022).

36. Dong Y, Brewer GJ. Global Metabolic Shifts in Age and Alzheimer's Disease Mouse Brains Pivot at NAD+/NADH Redox Sites. *Journal of Alzheimer's Disease : JAD* **71**, 119-140 (2019).
