# Supplementary material for: Metabolic phenotyping reveals an emerging role of ammonia abnormality in Alzheimer’s disease
Source: Nat Commun. 2024 May 7;15:3796. doi: 10.1038/s41467-024-47897-y (PMC11076546; doi:10.1038/s41467-024-47897-y)
Supplement: Supplementary file 2 — Reporting Summary [file 41467_2024_47897_MOESM2_ESM.pdf]

nature portfolio

Corresponding author(s): Qihao Guo, Fang Xie, Wei Jia

Last updated by author(s): Feb 20, 2024

Reporting Summary

Nature Portfolio wishes to improve the reproducibility of the work that we publish. This form provides structure for consistency and transparency in reporting. For further information on Nature Portfolio policies, see our Editorial Policies and the Editorial Policy Checklist.

Statistics

For all statistical analyses, confirm that the following items are present in the figure legend, table legend, main text, or Methods section.

n/a	Confirmed	
☐	☒	The exact sample size (*n*) for each experimental group/condition, given as a discrete number and unit of measurement
☐	☒	A statement on whether measurements were taken from distinct samples or whether the same sample was measured repeatedly
☐	☒	The statistical test(s) used AND whether they are one- or two-sided<br>*Only common tests should be described solely by name; describe more complex techniques in the Methods section.*
☐	☒	A description of all covariates tested
☐	☒	A description of any assumptions or corrections, such as tests of normality and adjustment for multiple comparisons
☐	☒	A full description of the statistical parameters including central tendency (e.g. means) or other basic estimates (e.g. regression coefficient) AND variation (e.g. standard deviation) or associated estimates of uncertainty (e.g. confidence intervals)
☐	☒	For null hypothesis testing, the test statistic (e.g. *F*, *t*, *r*) with confidence intervals, effect sizes, degrees of freedom and *P* value noted<br>*Give P values as exact values whenever suitable.*
☒	☐	For Bayesian analysis, information on the choice of priors and Markov chain Monte Carlo settings
☒	☐	For hierarchical and complex designs, identification of the appropriate level for tests and full reporting of outcomes
☐	☒	Estimates of effect sizes (e.g. Cohen's *d*, Pearson's *r*), indicating how they were calculated

Our web collection on statistics for biologists contains articles on many of the points above.

Software and code

Policy information about availability of computer code

Data collection	TMBQ 1.0 was used for metabolomics data collection; SPM12 and CAT 12 were ued for PET data collection.
Data analysis	Data analyses were performed using R (V3.5.1), GraphPad (V9.3), and STATA (V13.0).

For manuscripts utilizing custom algorithms or software that are central to the research but not yet described in published literature, software must be made available to editors and reviewers. We strongly encourage code deposition in a community repository (e.g. GitHub). See the Nature Portfolio guidelines for submitting code & software for further information.

Data

Policy information about availability of data

All manuscripts must include a data availability statement. This statement should provide the following information, where applicable:

- Accession codes, unique identifiers, or web links for publicly available datasets
- A description of any restrictions on data availability
- For clinical datasets or third party data, please ensure that the statement adheres to our policy

Metabolomics datasets of ADNI cohort can be accessed via the AD Knowledge Portal (https://adknowledgeportal.org; accession No. syn31513378 [https://www.synapse.org/#!Synapse:syn31513378]). The full complement of clinical and demographic data for the ADNI cohort are hosted on the LONI data sharing platform and can be requested at http://adni.loni.usc.edu/data-samples/access-data/. Metabolomics datasets of C-PAS (accession No. MTBLS4554 [https://www.ebi.ac.uk/metabolights/editor/MTBLS4554]) and ROSMAP (accession No. MTBLS9583 [https://www.ebi.ac.uk/metabolights/editor/MTBLS9583]) cohorts are accessible at MetaboLights. Source data are provided with this paper.

Research involving human participants, their data, or biological material

Policy information about studies with human participants or human data. See also policy information about sex, gender (identity/presentation), and sexual orientation and race, ethnicity and racism.

Reporting on sex and gender	sex were considered in this study.
Reporting on race, ethnicity, or other socially relevant groupings	all participants of this study are Chinese Han
Population characteristics	The mean age of the participants was 66.2 years (standard deviation = 8.6), with 41.3% of them being younger than 65 years.
Recruitment	All study participants were recruited from the Chinese Preclinical Alzheimer's Disease Study (C-PAS) cohort. Plasma samples and related information were obtained from 1397 individuals enrolled in the Chinese Preclinical Alzheimer's Disease Study (C-PAS) from April 2019 to June 2021. C-PAS is a nationwide longitudinal study aimed at identifying biomarkers for early detection and progression tracking of Alzheimer's disease (AD). Inclusion and exclusion criteria, clinical and neuroimaging protocols, and other information about C-PAS are described in the manuscript and SI. All the participants are Chinese Han and sex/age/APOE stratification analysis were conducted and related results were provided.<br>Datasets of ADNI and ROSMAP cohorts were used for main results validation. These datasets were from our published papers (provided as references 5,8,9,10,15 in the manuscript) and the participant recruitment was described there in detail.<br>No bias on participants recruitment steps.
Ethics oversight	The ethics committee of Shanghai Sixth People's Hospital Affiliated to Shanghai Jiao Tong University School of Medicine reviewed and approved this study (2019-032), following the principles of the Declaration of Helsinki. ADNI is a multi-center study focused on biomarker development for AD detection and tracking. Informed consent was obtained from participants, and the study was approved by each participating site's institutional review board. The ROSMAP study was approved by the review board of Rush university, and participants provided informed consent.

Note that full information on the approval of the study protocol must also be provided in the manuscript.

Field-specific reporting

Please select the one below that is the best fit for your research. If you are not sure, read the appropriate sections before making your selection.

[x] Life sciences [ ] Behavioural & social sciences [ ] Ecological, evolutionary & environmental sciences

For a reference copy of the document with all sections, see nature.com/documents/nr-reporting-summary-flat.pdf

Life sciences study design

All studies must disclose on these points even when the disclosure is negative.

Sample size	GPower was used for sample size predetermination. The sample size should be larger than 30/group, when power was set as 0.8, alpha was set as 0.05, effect size was set as 0.5, for t-test, ANOVA, or Mann-Whitney tests (two tails). Our sample size/group is much larger than the predetermined number in full and stratified analysis.
Data exclusions	Outliers were identified through Cauchy distribution robust fit (K sigma=7). Any outliers (<0.2%) and missing values (<0.1%) were substituted using multivariate normal imputation.
Replication	the study data were from C-PAS cohort and the main results were validated by ADNI, and ROSMAP cohorts. The 3 cohorts are based on different projects from different countries and are ndependent.
Randomization	1) sex/age/APOE stratification analysis were conducted and related results were provided in the manuscript. 2) logistic regression and linear regression with adjustment of covariates were used for potential biomarker identification.

Blinding | This is a retrospective study. Investigators for participant recruitment and data collection were blinded to subsequent analysis. Investigators for measurement were blinded to diagnosis and pathological data. The instrument running order was randomized. The investigators for data analysis were blinded to the data collection and pretreatment. They knew the group allocation after raw data pretreatment.

Reporting for specific materials, systems and methods

We require information from authors about some types of materials, experimental systems and methods used in many studies. Here, indicate whether each material, system or method listed is relevant to your study. If you are not sure if a list item applies to your research, read the appropriate section before selecting a response.

Materials & experimental systems

n/a	Involved in the study	
☒	☐	Antibodies
☒	☐	Eukaryotic cell lines
☒	☐	Palaeontology and archaeology
☒	☐	Animals and other organisms
☐	☒	Clinical data
☒	☐	Dual use research of concern
☒	☐	Plants

Methods

n/a	Involved in the study	
☒	☐	ChIP-seq
☒	☐	Flow cytometry
☒	☐	MRI-based neuroimaging

Clinical data

Policy information about clinical studies

All manuscripts should comply with the ICMJEguidelines for publication of clinical research and a completedCONSORT checklist must be included with all submissions.

Clinical trial registration	NA
Study protocol	NA
Data collection	NA
Outcomes	NA
